# The Impact of Staff Turnover and Staff Density on Treatment Quality in a Psychiatric Clinic

**DOI:** 10.3389/fpsyg.2016.00457

**Published:** 2016-03-31

**Authors:** Wolfram A. Brandt, Christoph J. Bielitz, Alexander Georgi

**Affiliations:** ^1^Sigma TagesklinikBad Säckingen, Germany; ^2^Sigma-Zentrum für AkutmedizinBad Säckingen, Germany

**Keywords:** psychiatry, psychotherapy, health expenditures, staff turnover, staff density, staff retention

## Abstract

Intuition suggests that improving stability of the health workforce brings benefits to staff, the organization and, most importantly, the patients. Unfortunately, there is limited research available to support this, and how health workforce stability can contribute to reduced costs and better treatment outcomes. To help to rectify this situation, we investigated the effects of staff turnover and staff density (staff members per patient) on the treatment outcome of inpatients in a psychiatric clinic. Our data come from the standard assessment of 1429 patients who sought treatment in our clinic from January 2011 to August 2013. Correlation analysis shows no significant effect of raw staff turnover (the total number of psychiatrists, physicians and psychologists starting or quitting work per month) on treatment quality. However, we do find two significant beneficial effects: first, a higher staff consistency (time without staff turnover) and second, a higher staff density lead to an improvement of treatment quality. Our findings underline the dire need for an extended effort to achieve optimal staff retention, both to improve patient’s outcomes and to reduce health expenses.

## Introduction

Staff turnover has been identified as a factor contributing to direct loss of revenue due to associated cost (e.g., searching for a new employee, job interviews, severance pay, administrative costs, training costs) and to more indirect costs resulting from factors like varying customer loyalty, possible loss of expertise, increase of error rate, increase of occupational accidents, resources tied by increased need for knowledge management and loss of product quality (Karsan, 2007; Ton and Huckman, 2008; Hausknecht et al., 2009; Hancock et al., 2013; Koerdt, 2014).

For the customer the individual impact from loss of product quality ranges from being simply annoying (e.g., imperfectly aligned cookie halves) to being downright life-threatening (e.g., defective air bags) but has in any case consequences both on the microeconomic (e.g., by warranty cases) and the macroeconomic (e.g., by increasing national and international costs for safety and occupational health) level (Harrington, 1987, 1999; Roth and Albright, 1994; Suhrcke et al., 2006b).

As an equivalent to what is called “product” in economy we consider the outcome also called treatment quality in healthcare systems. Since, by definition, the services of healthcare institutions always deal with health, life and death, the assurance of the highest possible product quality (i.e., effectiveness of treatment) is not merely an economical obligation (Suhrcke et al., 2006a,b, 2008) but an ethical duty.

Turning more specifically to the healthcare domain, there is surprisingly little research exploring the impact of staff turnover on effectiveness of treatment (Buchan, 2010), a notable exception being the increasing body of evidence that nursing staff turnover in nursing homes results in reduced quality of care (Castle and Engberg, 2005; Bostick et al., 2006; Van Bogaert et al., 2013).

Considerably less extensive is the body of research on the impact of staff turnover on outcome quality in psychiatric, psychosomatic and psychotherapeutic clinics (Ruff and Werner, 1988; Williams and Potts, 2010), with the existing studies focusing on structural aspects of treatment quality [e.g., the difficulties with the implementation of treatment protocols (Woltmann et al., 2008)]. This may be for a variety of reasons, beginning with the difficulty of defining outcome quality (Rehmann-Sutter, 2009) and ending in the possibility of misuse of outcome data resulting in organizational stigma (Lilford et al., 2004). Nevertheless, outcome quality is the factor which has the highest priority for the patients, which expect to be healed or at least to have their symptoms alleviated. The research examining the effects of personnel stability on patients’ outcomes in psychiatric clinics is very scarce contrary to the increasing body of research examining nursing homes. Therefore it is crucial to extend our knowledge on factors influencing psychiatric outcome quality, both to be able to offer the best possible treatment to our patients and to support qualified decision-making on the organizational and political level (Buchan, 2010).

In our study we define outcome quality as the reduction of symptom severity over the course of treatment. We argue that, since individual therapists differ in efficacy (Wampold and Brown, 2005), so do therapeutic teams. In our clinic each staff team consists of psychologists, psychiatrists, physicians, nurses, sport therapists, social workers, therapists for various non-verbal therapies (e.g., music therapy, bodywork therapy, expressive arts therapy, dance therapy) and occupational therapists, many of them working daily with their patients. The core treatment consists of psychiatric treatment by the psychiatrists, somatic treatment by the physicians and individual and group psychotherapy by the psychologists plus individual additional treatment by the other therapists of our clinic.

We were interested in finding out to what extent changes within staff consistency affected the outcome of patients treated during times of change compared to times of stability. In general, experienced staff left for various reasons (both voluntary and involuntary) and were replaced by new, less experienced staff who spent several weeks adjusting to the new job. Whatever the experience level of staff leaving and arriving was, we anticipated that changes would reduce cohesion within the staff team and coherence on the organizational level resulting in a negative impact on outcome (McAlearney et al., 2013).

## Materials and Methods

### Sample Description

The sample consists of *N* = 1429 consecutively admitted inpatients (collected at Sigma-Klinik, Bad-Säckingen, Germany) over a period from January 2011 to August 2013. Patients gave us written informed consent for our analyses; the capacity to consent was determined by an extensive interview by both the head psychiatrist and the responsible psychologist following the guidelines of our quality management system (certified after DIN EN ISO 9001:2008). Our strictly non-interventional study has been approved to conform the applicable legal and ethical guidelines by our internal review board belonging to the Sigma-Zentrum für Akutmedizin, Bad Säckingen, Germany.

The mean age was 50 years (*SD* = 12; range 18–89 years; quartiles 25% = 43.5; 50% = 51.5; 75% = 58.1). 50.9% (*n* = 727) were female, 49.1% (*n* = 702) were male.

Mean duration of treatment was 58.5 ± 32.2 days (quartiles 25% = 34; 50% = 55; 75% = 76).

The distribution of primary diagnoses [ICD-10 Chapter V(F), (World Health Organization, 1993)] is summarized in **Table 1**.

**Table 1:** Distribution of primary ICD-10-diagnoses in our sample.

Diagnosis [ICD-10, Chapter V(F)]	*n*	%
F3: Affective disorders	1023	71.6%
F4: Neurotic, stress-related and somatoform disorders	253	17.7%
F6: Disorders of adult personality and behavior	63	4.4%
F2: Schizophrenia, schizotypal and delusional disorders	31	2.2%
F5: Behavioral syndromes associated with physiological disturbances and physical factors	26	1.8%
F1: Mental and behavioral disorders due to psychoactive substance use	14	1.0%
Other (F0, F7, F8, F9)	19	1.3%

Occupancy (total number of patients treated per month) ranges from minimum = 96 to maximum = 122 (*M* ±*SD* = 113.9 ± 5.85; quartiles 25% = 111; 50% = 116; 75% = 118).

Basic treatment consist of daily psychiatrist’s and physician’s visits, three sessions a week of individual psychotherapy and three sessions a week of group psychotherapy. In addition every patient receives a personalized treatment plan tailored to his or her needs (e.g., biofeedback, social training, sport-therapy, psychopharmacological treatment etc.) and individual care by our nursing staff. In every case, treatment follows the applicable national German clinical practice guidelines (German Association of the Scientific Medical Societies (AWMF) – Standing Guidelines Commission, 2012).

The total number of psychiatrists, psychologists and physicians per month ranges from minimum = 43 to maximum = 46 (*M* ±*SD* = 44.6 ± 1.0; quartiles 25% = 44; 50% = 44; 75% = 46). Many of our employees (63%) are working part-time (work time ranges from minimum = 50% to maximum = 100%; quartiles 25% = 70%; 50% = 80%; 75% = 100%).

### Measures

Routine assessment includes several self-rating questionnaires and standardized clinical assessments which are applied pre- and post-treatment.

For our study we use the results from the SCL-90 (Franke, 2002) “Global Severity Index” (GSI) scale. The GSI is designed to measure overall psychological distress and allows assessment of symptom severity without being restricted to specific disorders and therefore allows the comparison of treatment quality in a heterogeneous sample (Hessel et al., 2001).

Staff turnover, staff density and staff consistency were calculated using employment records.

### Hypotheses and Data Analysis

We tested the following hypotheses:

(1) Effect of treatment: Treatment in our clinic decreases symptom severity in our patients significantly. Although apparently trivial, this is the basis for the following hypotheses.
(2) Variation over time: Treatment quality varies significantly over time. Another basic question: If there is no detectable variation, there can be no factors causing variation.
(3) Seasonal effects: The variation in treatment quality is not explainable by seasonal effects.
(4) Gender, age, duration of treatment and occupancy: Treatment quality is not associated with gender, age, duration of treatment or occupancy.
(5) Staff density: A higher staff density is associated with a higher treatment quality.
(6) Staff turnover: A higher staff turnover is associated with a lower treatment quality.
(7) Staff consistency: A higher staff consistency (consecutive time without turnover) is associated with a higher treatment quality.

Analysis was done using SPSS 21 for Windows. We use non-parametric statistics with Monte-Carlo simulations based on 10^7^ samples wherever necessary (i.e., because of scale restrictions, sample size or unmet assumptions of normality), otherwise we use parametric statistics. Due to the exploratory nature of our study, we omitted corrections for multiple testing.

## Results

### Effect of Treatment

In order to assess the effectiveness of our treatment we compared the pre-treatment GSI scores with the post-treatment GSI scores (delta-GSI). We found a significant reduction of symptom severity after treatment [*M*_pre_ ±*SD*_pre_ = 67.5 ± 8.4 and *M*_post_ ±*SD*_post_ = 53.9 ± 10.6; paired sample *t*-test (*t* = 52.14, df = 1428), *p* < 0.0001]. The outcome was an effect size (Cohen’s *d*, Cohen, 1988) of *d* = 1.38 (CI_95%_ = 1.31–1.45).

### Variation Over Time

In order to assess the significance of the changes in treatment quality over time we calculated a Kruskal–Wallis test with point of time (year and month of start of treatment) as factor and delta-GSI (quality of treatment) as dependent variable.

We found a significant change of quality of treatment over time [χ^2^(df = 31, *N* = 1429) = 48.99; *p* = 0.020].

### Seasonal Effects

To assess possible seasonal effects in quality of treatment, we calculated a time-series analysis (SPSS Module “Forecasting,” “Expert Modeler”). The resulting ARIMA-Model is not better than the simple means-model (Stationary *R*^2^= *R*^2^= 1.04^∗^10^-13^).

We conclude that there is no seasonal effect for quality of treatment [Ljung-Box Q(18) = 19.5; *p* = 0.361] in our sample.

### Gender, Age, Duration of Treatment, and Occupancy

We do not find a significant association between gender [*M*_female_ ±*SD*_female_ = 13.75 ± 9.98 and *M*_male_ ±*SD*_male_ = 13.36 ± 9.67; independent sample *t*-test (*t* = -0.75, df = 1427), *p* = 0.45], age (Spearmans ρ = 0.027; *p* = 0.3), duration of treatment (ρ = -0.032; *p* = 0.222) or occupancy (total number of patients) (ρ = -0.036; *p* = 0.173) and quality of treatment.

### Staff Density

We define staff density as the quotient (Number of psychiatrists + Number of physicians + Number of psychologists)/Number of patients. Staff density ranges from minimum = 0.36 to maximum = 0.48 (quartiles 25% = 0.38; 50% = 0.39; 75% = 0.40).

We found a significant association between staff density and quality of treatment (Spearmans ρ = 0.055; *p* = 0.037).

### Staff Turnover

We define staff turnover as the total number of psychiatrists, physicians and psychologists starting or quitting work per month. Staff turnover ranges from minimum = 0 to maximum = 4 (quartiles 25% = 0; 50% = 1; 75% = 2).

We found no significant association between staff turnover and quality of treatment (Spearmans ρ = -0.003; *p* = 0.909).

### Staff Consistency

We define staff consistency as the total number of consecutive months without any staff turnover preceding the start of treatment. Staff consistency ranges from minimum = 0 to maximum = 5 (quartiles 25% = 0; 50% = 0; 75% = 1).

We found a significant association between staff consistency and quality of treatment (Spearmans ρ = 0.068; *p* = 0.01).

### Combined Model

**FIGURE 1:**
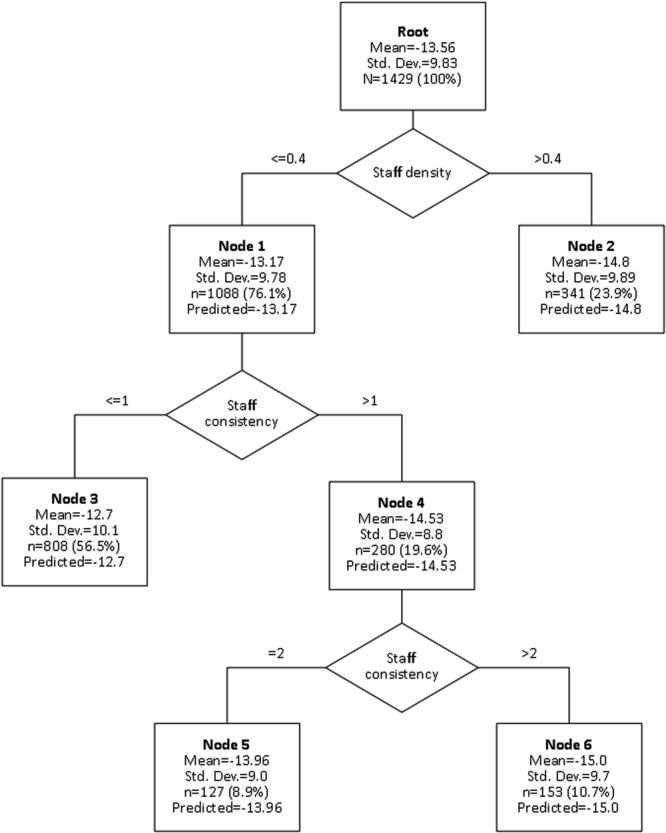
**Combined model including staff density, staff consistency and resulting treatment quality**.

In order to combine the previously identified variables in a simple model making no assumptions about linearity or distribution, we used the CART-algorithm. To prevent overfitting we restricted the maximum tree depth to 5, the minimum end-node size to 50 and the minimum change in improvement for each node to 0.05. Validation was done using a 10-fold sample cross-validation. The resulting tree is shown in **Figure 1**.

To estimate the differences between the single nodes, we calculated pairwise Mann–Whitney-*U*-tests for all combinations. The resulting *p*-values are summarized in **Table 2**.

**Table 2:** *p*-Values of the differences between the subgroups of the combined model.

Node	2	3	5	6
2	*–*	0.001^∗^	0.506	0.928
3	0.001^∗^	–	0.075	0.004^∗^
5	0.506	0.075	–	0.563
6	0.928	0.004^∗^	0.563	–

*^∗^*p* < 0.01.*

To estimate the differential effect sizes for treatment in the subgroups, we calculated Cohen’s *d* with 95% confidence interval. The results are summarized in **Table 3**.

**Table 3:** Effect sizes for treatment in the subgroups of the combined model.

Node	Cohen’s *d* (95% CI)
2	1.496 (1.34–1.65)
3	1.263 (1.17–1.36)
5	1.55 (1.29–1.81)
6	1.73 (1.48–1.98)

## Discussion

We are not surprised to find a variation of treatment quality over time in a naturalistic setting. This variation is not explainable by seasonal effects, occupancy, gender or duration of treatment. Nor do we find an association between treatment quality and raw staff turnover. In order to analyze more precisely the impact of the factors mentioned above, a larger sample (ideally) from several independent clinics would be necessary.

What we do find are two effects: one is an effect of staff consistency (defined as the consecutive time without staff turnover) while the other is an effect of staff density (defined as the mean number of core therapists per patient) on treatment quality. Although being numerically relatively small (from ρ = 0.068 to ρ = 0.055, respectively), these effects are strong enough to become detectable on an institutional level. In our exploratory combined model (**Figure 1**, **Tables 2** and **3**), we see that the lowest treatment quality is achieved in times of both low staff consistency and low staff density (node 3, *d* = 1.263) and the best results in times of high staff consistency (node 6, *d* = 1.73) or high staff density (node 2, *d* = 1.496).

These results are in line with the findings of Park and Shaw (2013), who find a detrimental effect of staff turnover and staff reduction on organizational performance in their large meta-analysis, and Abel et al. (2012), who find an enhanced productivity linked to the density of human capital.

The beneficial effect of a higher staff density on treatment quality can be explained for instance by the lower cost of generating new ideas and exchanging information between different experts and different occupational groups (“knowledge spillover”). Especially the flow of knowledge is increased by the amount of personal interaction and face to face contacts that people experience. This form of contact has been shown to enhance productivity when information is imperfect, rapidly changing, or not easily codified (Storper and Venables, 2004) – key features of personalized psychiatry and psychotherapy. It also means that the archaic angst of competing local companies and the associated potential loss of profit are ill-founded, since the synergetic benefits of knowledge-assimilation and -transfer outweigh potential losses.

The beneficial effect of a higher staff consistency on treatment quality is probably more complex. We argue that, since one of the most important base variables of psychotherapy is the therapeutic relationship (Grawe, 2005), treatment quality suffers significantly if therapists experience an atmosphere of lowered relationship safety and therefore are less able to offer a secure therapeutic relationship themselves.

Our findings support the value of the concept of “Organizational Coherence” with the intercorrelating components “People,” “Processes’ and “Perspectives” by McAlearney et al. (2013). In this model, a coherent organization has the shared perspective that employees should be respected, can be trusted, and that they can change and learn. They have individuals acting as champions and change agents able to drive improvement efforts and maintain coherence in the face of organizational confusion. Also there is a culture that values negotiated agreements rather than imposition of others’ values and beliefs upon employees (component “People”). In the second component “Processes,” coherent organizations have a balance between top–down and bottom–up processes to encourage engagement within the organization and a flexibility to manage and organize as needed to achieve shared goals. There is consistency among project efforts rather than a multitude of unconnected projects or goals. The third component “Perspectives” includes an organizational orientation toward forming long-term relationships and enabling sense-making to permit a long-term commitment to improvement. In contrast to organizations suffering from political power-plays or an abundance of short-term objectives, this allows for an alignment of the organization’s agenda with the goals and objectives of individuals, groups, and other units within the organization and is critical in helping the organization to move forward to achieve long-term success.

A lowered staff consistency results in a disruption of all three components (people, process and perspective) and is therefore potentially detrimental to organizational performance.

One important limitation of our study is the fact that we analyze our data *post hoc* which means we can only make assumptions about the direction of causality for our associations. Another (necessary) limitation is our focus on therapeutic personnel providing the core treatment (i.e., psychiatrists, psychologists, and physicians) disregarding the effect of individual treatment plans and nursing staff care. This is part of our naturalistic design, although we tried to control for the possible influence of gender, age, duration of treatment and seasonal effects on treatment quality by testing for it and thereby increasing internal validity. We also consider all personnel as equal, disregarding differing levels of experience and reasons of quitting or starting work in our clinic. This is one possible reason for our numerically relatively small effects since Park and Shaw (2013) report large detrimental effects of staff turnover for voluntary (ρ = -0.15) and reduction-in-force (ρ = -0.17) turnover whereas involuntary turnover has only a small to negligible effect (ρ = -0.01).

Another limitation is the proximal nature of our measures influencing treatment quality. Since both staff turnover and staff density depend on a variety of organizational, managerial and inter- and intrapersonal variables (e.g., experience, communication protocols, job satisfaction, morale etc.) we can only speculate about the underlying processes and determinants affecting the quality of treatment.

Nevertheless there are important implications for the support of staff in healthcare organizations. Although change is seen as a constant in hospitals and other healthcare services, the associated costs are far from being negligible. Waldman et al. (2004) calculate the turnover costs as at least 5% of the annual operating budget of a given healthcare organization, not including the costs of decrements of treatment quality. For illustration purposes: if these numbers could be transferred to german national levels, the costs for staff turnover alone would amount to 1.4 billion Euros (≈1.41 billion US-dollars) per year just for psychiatric disorders (Statistisches Bundesamt (Destatis), 2010).

## Conclusion

Our findings show that contextual factors like reduced staff density and stability impact negatively on patient’s outcomes thus underlining the dire need for an extended effort to achieve optimal staff retention. The expected mid- to long-term cost reduction both for the single institution as well as for the direct (e.g., treatment costs) and indirect (e.g., disability costs) national health expenses are substantial. To assist both policy makers and managers in their decisions, expanding our knowledge on personnel factors impacting on treatment quality seems extremely important and well warrants further research.

## Author Contributions

WB and AG contributed equally to this work. The tasks consisted of data aggregation, data analysis and interpretation, literature research and formulating the initial draft of the manuscript. CB helped in the development of the manuscript in the submitted form and the interpretation of the data.

## Conflict of Interest Statement

The authors declare that the research was conducted in the absence of any commercial or financial relationships that could be construed as a potential conflict of interest.
